# Building infrastructure for outcomes-based agreements in Canada: can administrative health data be used to support an outcomes-based agreement in oncology?

**DOI:** 10.1007/s00520-022-07486-5

**Published:** 2022-12-13

**Authors:** Winson Y. Cheung, Chris Cameron, Arif Mitha, Allison Wills

**Affiliations:** 1 University of Calgary, Cancer Care Alberta, Oncology Outcomes, Calgary, AB Canada; 2 EVERSANA Life Science Services LLC, Chicago, IL USA; 3 20Sense, Toronto, ON Canada

**Keywords:** Knowledge transfer, Outcomes-based agreements, Real-world evidence, Health outcomes, Oncology

## Abstract

**Background:**

Outcomes-based agreements (OBAs) have the potential to provide more timely patient access to novel therapies, although they are not suitable for every new medication or reimbursement scenario. The authors of this paper studied how to operationalize an OBA in oncology by leveraging existing real-world data (RWD) infrastructure in the province of Alberta.

**Objective:**

The main objectives were to (1) evaluate which health outcomes in oncology are suitable for OBAs and whether they can be tracked with existing infrastructure, and (2) determine how RWD in oncology can be used to implement an OBA and the expected timing for delivery.

**Methods:**

Using the Oncology Outcomes (O2) Group infrastructure and Alberta administrative data, a review of five key oncology outcomes was performed to determine suitability to support an OBA.

**Results:**

Overall survival and time-to-next-treatment were determined as potentially suitable oncology outcomes for OBAs; progression-free survival, patient-reported outcomes, and return to work were deemed inadequate for OBAs at the current time due to data limitations.

**Conclusions:**

Results indicate that it is feasible to leverage RWD to support OBAs in oncology in Alberta, with minimal additional data, resources, and infrastructure. The operational processes and steps to collect and analyze RWD for OBAs were identified, starting with performing an RWD feasibility study. The expected timeframe to fulfill the real-world evidence (RWE) requirements for an OBA is approximately 3 years for cancers with short trajectories.

## Background

With the growing number of promising therapies with imperfect data, particularly in rare diseases and precision oncology, coupled with long reimbursement timelines, timely access to novel therapies has become increasingly challenging. Outcomes-based agreements (OBAs) have the potential to accelerate patients’ access to these therapies, while mitigating risk for payers [1, 2]. Traditionally, an OBA is an agreement between a manufacturer and a payer in which the manufacturer will issue a refund or rebate to the payer based on how well the therapy performs in a real-world patient population, measured against an agreed-upon, pre-defined set of benchmarks. OBAs are a strategy to address uncertainties that create access barriers; they are not a replacement for clinical trials, and they are not appropriate for all drugs or for all reimbursement scenarios.

The UK’s 2021 Commercial Framework for New Medicines advises that OBAs should “only be considered once simple discounts have been demonstrated to be unsuitable” [3]. Research conducted by the RWE & OBA Working Group, established in 2019 to explore the opportunity for RWE to support OBAs in Canada, suggests that OBAs offer the greatest benefit in the following circumstances [4]:

- *Variable response*: When clinical trials suggest that only a limited proportion of patients (e.g., 50%) reach a desired health outcome, OBAs can reduce a payer’s risk by limiting ongoing reimbursement to patients who meet agreed-upon outcome criteria.
- *Limited data*: When promising but incomplete early clinical trial data make it difficult to assess a drug’s performance, an OBA can provide access to patients with no suitable treatment alternatives.
- *Disputed therapeutic benefit*: When stakeholders disagree on the magnitude of therapeutic benefit suggested by clinical trial data, an OBA can help patients access treatment earlier, with continued access dependent on proof of benefit.

As detailed in Health Canada’s proposed national strategy for high-cost drugs for rare diseases, the success of an OBA depends on clear, objective measures of benefit [5]. If the evidence ultimately reveals a more modest benefit than anticipated, OBAs allow for reduction or discontinuation of reimbursement, thus mitigating risk for public and private payers.

OBAs have gained ground internationally as a strategy for managing access and risk, but are still in their infancy in Canada. Recognized barriers include inconsistent availability of appropriate real-world data (RWD), potential increase in administrative burden, challenges in reaching agreement about the adjudication process to assess efficacy, and a lack of resources and infrastructure to implement such agreements. At the same time, Canada’s RWD infrastructure has been advancing, particularly in Alberta, which has been developing advanced infrastructure, broad data capture, and resources to support oncology analytics. The Oncology Outcomes (O2) group, a consortium of oncology research and clinical leaders, is playing a key role in this regard.

Compounding the aforementioned barriers, OBAs are confidential in Canada, and there are no publicly available examples for stakeholders to learn from. This study sought to fill this information gap by describing a feasible process for operationalizing the RWE requirements for an OBA.

The objectives of the study were to evaluate which health outcomes are suitable for OBAs in oncology and to examine if such health outcomes can be tracked using existing infrastructure in Alberta and Patient Support Programs (PSP). The study also explored the use of OBA data processes to facilitate the data tracking required to support an OBA.

## Methods

The lead author and investigator of the analysis, Dr. Winson Cheung, is an academic researcher with a special interest in RWE. Dr. Cheung had the final input and authority in the interpretation of the findings and the content of this paper. The second author, Dr. Chris Cameron, is recognized as a global thought leader in health economics and outcomes research. The last two authors, Allison Wills and Arif Mitha, have expertise in outcomes-based agreement planning and implementation, and belong to the RWE & OBA Working Group, established in 2019 to explore the opportunity for RWE to support OBAs in Canada. Payers were not involved in the analysis at this stage and will be invited to comment on these findings at a future date. None of the authors received any direct compensation from industry for this work.

In 2019 and 2020, the RWE & OBA Working Group’s research and analysis led to the development of several tools to aid in the evaluation and implementation of OBAs, including a *7-step OBA implementation framework* [4] and a *Decision modelling tool: outcomes-based agreement vs. price discount contract* [6].

The 7-step OBA implementation framework was used to support the research on RWE for OBAs. The framework includes the following steps:

1. Determine if the drug is fit for OBA
2. Negotiate OBA and design program
3. Build OBA program
4. Enroll doctors and patients in program
5. Begin and monitor drug treatment
6. Report and adjudicate
7. Conduct annual review

The study investigators evaluated five health outcomes for suitability to support an OBA in oncology in Canada: (1) overall survival (OS), (2) time to next treatment (TTNT), (3) progression-free survival (PFS), (4) patient-reported outcomes (PROs), and (5) return to work.

The decision to include TTNT as an outcome of interest, in addition to the well-established OS and PFS, aligns with the increasing use of TTNT as a surrogate outcome in both clinical trials and RWE. A 2020 analysis of TTNT, in the context of cutaneous lymphoma, described TTNT as a useful surrogate endpoint for “duration of clinical benefit” that also accounts for patient tolerance and adherence [7]. A real-world analysis of 4729 advanced cancer patients, reported in the *Journal of Clinical Oncology* in 2016, concluded that “concordance of TTNT and OS for patients with biomarker-associated therapies validates the clinical utility of TTNT as a surrogate endpoint that can be assessed using EMR extracted data” [8].

It should be noted that the alignment of TTNT with PFS is not always perfect, and thus TTNT estimates may exceed the corresponding PFS estimates in settings that lead to delays in subsequent therapies [9].

The investigators also assessed whether health outcomes can be tracked by the O2 Group with existing infrastructure in Alberta and by PSP infrastructure at a national level. They outlined a process for using the O2 Group’s capabilities and Alberta administrative health data, and created two additional process designs that leveraged PSP infrastructure and data.

## Results

### Health outcomes suitable for OBAs in oncology

Two health outcomes, namely, OS and TTNT, were identified as suitable for the collection of RWD to support OBAs in oncology using administrative data (Table 1). These outcomes were deemed suitable based on criteria of data readiness (accessible for an OBA, complete, and accurate), data interpretation (health outcome is clear and simple), and data timeframe (can be collected in a reasonable timeframe). OS data are available through the Alberta Cancer Registry and Vital Statistics, have been used in published studies [2], and measure an objective event that can be easily and consistently interpreted. Diagnoses with relatively short anticipated OS timeframes could be suitable for an OBA. TTNT data are accessible through the Alberta Pharmaceutical Information Network (PIN) database containing all prescription data in the province for all payers and have been used in published studies [2, 10]. A coding algorithm must be developed to determine a specific treatment pattern, and data are generally accessible within 1 month of dispensing. Progression-free survival, patient-reported outcomes, and return to work were currently deemed not suitable outcomes due to data limitations. PROs have future potential for use in OBAs, but additional RWD collection activities would be required.

**Table 1:** Health outcomes data readiness, interpretation, and timeframes in Alberta

	Health outcome	Suitable for an OBA?	Data readiness Accessible for an OBA, complete and accurate	Data interpretation Health outcome is clear and simple	Data timeframe Can be collected in a reasonable timeframe
	1. Overall survival (OS)	Yes	• AB Cancer Registry and Vital Statistics • Data have been used in published studies	• Binary data point, easy to interpret	• 6-month time lag
	2. Time to next treatment (TTNT)	Yes	• AB PIN Database contains all Rx’s dispensed in AB (all payers) • Data have been used in published studies	• Algorithm required to ascertain specific treatment pattern	• 1-month time lag
	3. Progression-free survival (PFS)	No	• AB administrative data • Incomplete: the timing of tests is not standardized	• Interpretation of results recorded in data is not standardized	N/A
	4. Patient-reported outcomes (PROs)	No—future potential	• AB administrative data: ESAS, EQ5D Surveys • Incomplete: not consistently administered to all patients • No published studies	• EQ5D and ESAS are frequently included in HTA submissions	N/A
	5. Return to work	No	• Data not available	• Patient may choose not to return to work	N/A

### Data processes to operationalize RWD for OBAs in oncology

Three data processes were identified to operationalize RWD for OBAs in oncology: (1) administrative data, (2) administrative data with PSP support, and (3) PSP data (see Table 2). Three data elements are managed within each process: (1) data planning, (2) data capture, and (3) data analysis.

**Table 2:** Process design for data collection to support outcomes-based agreements

		Process 1 Administrative data	Process 2 Administrative data with PSP support	Process 3 PSP data
Data planning	Feasibility study	O2	O2	O2
Data capture	Patient registry	Administrative data	PSP	PSP
	drug distribution	Administrative data	PSP	PSP
	Health outcome data source	Administrative data	Administrative data	PSP
Data analysis	Analysis	O2	O2	O2

#### Data planning

When considering an OBA, an RWD feasibility study is advised. This can be conducted by either a manufacturer, a payer, or both, in collaboration with a data expert. The aim of the feasibility study is to determine if the appropriate health outcomes data can be collected to meet the needs of an OBA for a specific drug scenario. The feasibility study seeks to answer the following questions:

1. Which health outcomes measurements could be used for the drug of interest?
2. What RWD is available and what data sources are most appropriate to measure the identified health outcomes?
3. Regarding the identified health outcomes:
  - Does the data source have the required data quality to support the OBA?
  - Is the patient population sufficient to allow for the analysis of the outcomes?
  - What is the anticipated time to complete the data component of the OBA, including data capture and results generation?

The findings of the feasibility study should serve as a key input when payers and manufacturers are designing OBAs and can provide important insights on OBA design methodology. Feasibility discussions can happen early, including prior to drug launch. Ethics approval may be required to use certain data sets for the feasibility study and for the OBA.

#### Data capture

Elements that impact the way data are captured and populated in databases include (1) the patient registry, (2) drug distribution, and (3) the health outcomes data source. Depending on the scenarios for different drugs, three processes were developed to enable data capture for the purpose of measuring health outcomes for an OBA (Table 2).

*Process 1: Administrative data*: Administrative data refer to data that are populated during regular clinical or administrative processes. Process 1 provides an OBA strategy that uses only administrative data. For OS and TTNT, the benefits of this process include.

1. Administrative data are already being captured in regular clinical or administrative processes and populated in administrative data sources. No additional infrastructure or processes are required for the purposes of OBA data collection.
2. There is no additional work for doctors or patients for the data collection required for an OBA.

However, the use of only administrative data also has limitations: there are fewer controls for patient eligibility, which could lead to a wider scope of patients being prescribed the drug than intended within the OBA criteria. As such, the use of administrative data exclusively may not be appropriate for all OBAs, and additional data process options were designed (process 2 and process 3).

*Process 2: Administrative data and PSP infrastructure*: Process 2 combines administrative data with PSP infrastructure support. The PSP infrastructure is used to create a patient registry, which makes it possible to align patient eligibility with the OBA criteria. As in process 1, administrative data are also used as the health outcomes data source.

*Process 3: PSP data and infrastructure*: Process 3 uses PSP infrastructure for the patient registry and PSP data as the health outcome data source. The use of PSP infrastructure and data in OBAs offers several unique advantages compared to administrative data:

1. The use of a PSP registry allows for greater oversight and control of patient eligibility.
2. If the PSP is used for all data capture activities (process 3), it can potentially be scaled to the national level and include all payers.
3. The PSP is flexible and can be customized to meet the needs of the OBA.

However, the use of PSP infrastructure for OBA data collection would entail additional work for doctors and patients. Also, PSPs need to be strategically set up at the outset to capture data to support a potential OBA. A key consideration is that the PSP must enable the collection of high-quality data that meet the requirements of payers and manufacturers.

#### Data analysis

Data should be accessed and analyzed by data experts, with results aggregated and reported as specified in the OBA. This includes PSP data, which can be analyzed by a third-party data expert such as the O2 group.

### Total estimated time required for the RWD component of an OBA—2.5 to 3 years

**Fig. 1:**
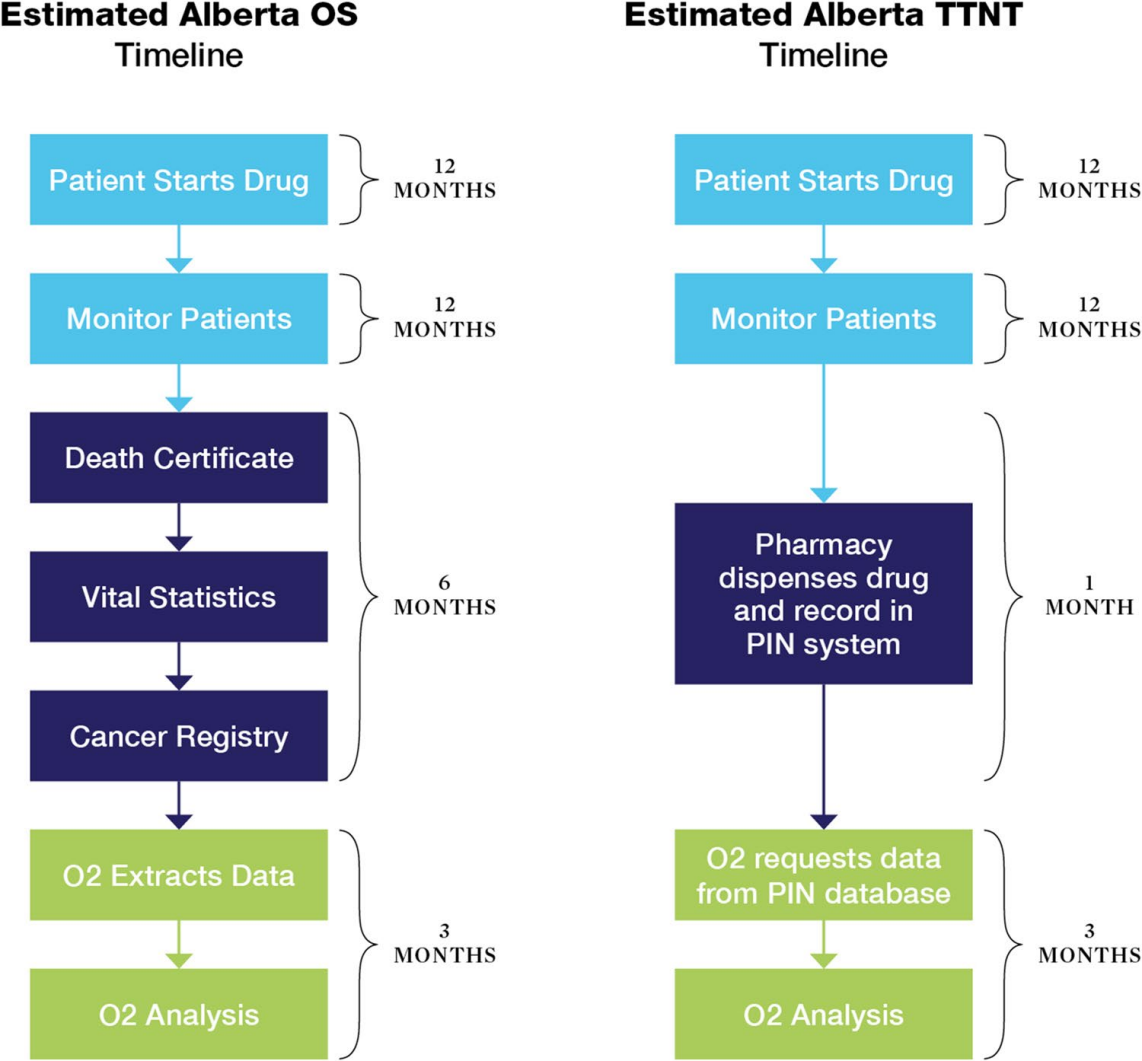
Estimated time required for the RWD component of an OBA in a hypothetical scenario

The total estimated timeframe required to complete the RWD component of an OBA is 2.5–3 years when using OS or TTNT in Alberta (Fig. 1). For both OS and TTNT, patients are recruited over a 12-month period and then monitored for an additional 12 months after initiation of therapy.

For OS using process 1 (see Table 2, Fig. 1), the following estimated timeline was developed: 12 months for a sufficient number of patients to start on drug; 12 months to monitor patients; 6 months for data collection logistics to receive information on survival status; 3 months for analysis and reporting by a team with data expertise. This resulted in a total approximate timeframe of 2 years and 9 months. This timeframe aligns with the oncology OBA process presented in an Australian study [11]. For TTNT using process 1 (see Table 2, Fig. 1), the following estimated timeline was developed: 12 months for a sufficient number of patients to start on drug; 12 months to monitor patients; 1 month for data collection logistics in the pharmacy PIN system; 3 months for analysis and reporting by a team with data expertise. This resulted in a total approximate timeframe of 2 years and 4 months.

## Discussion

This research has found that it is feasible to operationalize an OBA in oncology using Alberta administrative health data with minimal additional data resources, although administrative support to conduct the analyses would still be required. For stakeholders exploring the value of OBAs for a novel medication, this analysis can serve as a “proof of concept” model that demonstrates feasibility under some circumstances.

The health outcomes of OS and TTNT, which are already being collected in Alberta, were found to be suitable to support an OBA, with data management support from the O2 group. Three operational processes to collect, analyze, and report RWD for OBAs were identified, using administrative health data, PSP infrastructure, and PSP data. All processes start with an RWD feasibility study conducted by a team with data expertise. An estimated timeframe to conduct the RWD component of an OBA is 2.5–3 years.

Insights from this research may be valuable to the Canadian Agency for Drugs and Technologies in Health (CADTH) and Institut national d'excellence en santé et en services sociaux (INESSS), which provide research and analysis to support decisions about new therapies. Manufacturers, provincial drug plans, and private insurers that are considering implementing OBAs in oncology may also benefit from the findings shared here. When exploring the feasibility of OBAs, it will be important for payers and other stakeholders to quantify and manage the administrative burden to ensure it does not cancel the benefits of OBAs.

## Conclusions

The findings from this study should enhance Canadian stakeholders’ understanding of how an OBA in oncology can be operationalized using administrative data and the time it will take to complete an OBA using existing RWD infrastructure.

It should be noted that this analysis addresses only one barrier to OBA implementation, namely, the availability of appropriate RWD. Further research is needed to explore and address additional potential barriers such as administrative burden and efficacy of adjudication processes. Future research should also focus on how OBAs can be operationalized at a pan-Canadian level and in therapeutic areas outside oncology, such as rare diseases.

## Data Availability

Aggregate-level data analysed in this study are available on request from the corresponding author. Individual-level data are not publicly available due to Canadian data privacy laws governing personal health information.

## Abbreviations

*OBAs*: Outcomes-based agreements
*RWD*: Real-world data
*RWE*: Real-world evidence
*O2 Group*: Oncology Outcomes (O2) group
*PSP*: Patient support programs
*OS*: Overall survival
*TTNT*: Time to next treatment
*PFS*: Progression-free survival
*PROs*: Patient-reported outcomes
*PIN*: Alberta Pharmaceutical Information Network
*CADTH*: Canadian Agency for Drugs and Technologies in Health
*INESSS*: Institut national d’excellence en santé et en services sociaux
